# Supplementary material for: Modeling glioblastoma heterogeneity as a dynamic network of cell states
Source: Mol Syst Biol. 2021 Sep 16;17(9):e10105. doi: 10.15252/msb.202010105 (PMC8444284; doi:10.15252/msb.202010105)
Supplement: Supplementary file 6 — Source Data for Figure 5 [file MSB-17-e10105-s004.zip › Figure5A_sourcedata/GSEA_3071/hallmarks_stateIII.GseaPreranked.1623742540963/HALLMARK_ESTROGEN_RESPONSE_LATE.html]

Details for gene set HALLMARK\_ESTROGEN\_RESPONSE\_LATE[GSEA]

|  || Dataset | state33071 |
| Phenotype | NoPhenotypeAvailable |
| Upregulated in class | na\_neg |
| GeneSet | HALLMARK\_ESTROGEN\_RESPONSE\_LATE |
| Enrichment Score (ES) | -0.35402498 |
| Normalized Enrichment Score (NES) | -1.9148341 |
| Nominal p-value | 0.0031982942 |
| FDR q-value | 0.011079952 |
| FWER p-Value | 0.124 |
Table: GSEA Results Summary

  

Fig 1: Enrichment plot: HALLMARK\_ESTROGEN\_RESPONSE\_LATE      
 Profile of the Running ES Score & Positions of GeneSet Members on the Rank Ordered List

  

| PROBE | GENE SYMBOL | GENE\_TITLE | RANK IN GENE LIST | RANK METRIC SCORE | RUNNING ES | CORE ENRICHMENT || 1 | MOCS2 |  |  | 303 | 0.290 | -0.0647 | No |
| 2 | FKBP5 |  |  | 657 | -0.263 | -0.1435 | No |
| 3 | ALDH3B1 |  |  | 822 | -0.270 | -0.1733 | No |
| 4 | SLC2A8 |  |  | 950 | -0.277 | -0.1932 | No |
| 5 | KLF4 |  |  | 973 | -0.278 | -0.1860 | No |
| 6 | CPE |  |  | 1081 | -0.284 | -0.2005 | No |
| 7 | RNASEH2A |  |  | 1103 | -0.285 | -0.1927 | No |
| 8 | TFPI2 |  |  | 1128 | -0.286 | -0.1857 | No |
| 9 | TIAM1 |  |  | 1276 | -0.294 | -0.2100 | No |
| 10 | ATP2B4 |  |  | 1347 | -0.297 | -0.2143 | No |
| 11 | JAK2 |  |  | 1401 | -0.300 | -0.2141 | No |
| 12 | DNAJC12 |  |  | 1510 | -0.306 | -0.2277 | No |
| 13 | TOB1 |  |  | 1573 | -0.310 | -0.2293 | No |
| 14 | MICB |  |  | 1576 | -0.311 | -0.2155 | No |
| 15 | PLK4 |  |  | 1642 | -0.315 | -0.2177 | No |
| 16 | GAL |  |  | 1679 | -0.317 | -0.2123 | No |
| 17 | ID2 |  |  | 1780 | -0.323 | -0.2231 | No |
| 18 | NAB2 |  |  | 1927 | -0.331 | -0.2454 | No |
| 19 | SLC16A1 |  |  | 2012 | -0.336 | -0.2515 | No |
| 20 | XBP1 |  |  | 2097 | -0.341 | -0.2574 | No |
| 21 | PLXNB1 |  |  | 2206 | -0.349 | -0.2691 | No |
| 22 | WFS1 |  |  | 2307 | -0.358 | -0.2783 | No |
| 23 | IMPA2 |  |  | 2379 | -0.363 | -0.2798 | No |
| 24 | CD44 |  |  | 2471 | -0.371 | -0.2861 | No |
| 25 | KIF20A |  |  | 2561 | -0.379 | -0.2915 | No |
| 26 | DHCR7 |  |  | 2654 | -0.386 | -0.2973 | No |
| 27 | EMP2 |  |  | 2695 | -0.390 | -0.2896 | No |
| 28 | SIAH2 |  |  | 2946 | -0.414 | -0.3349 | Yes |
| 29 | SCARB1 |  |  | 2975 | -0.417 | -0.3228 | Yes |
| 30 | SLC1A4 |  |  | 3051 | -0.426 | -0.3224 | Yes |
| 31 | CHPT1 |  |  | 3060 | -0.426 | -0.3048 | Yes |
| 32 | BCL2 |  |  | 3081 | -0.429 | -0.2901 | Yes |
| 33 | SGK1 |  |  | 3150 | -0.437 | -0.2874 | Yes |
| 34 | FAM102A |  |  | 3271 | -0.454 | -0.2973 | Yes |
| 35 | MEST |  |  | 3363 | -0.473 | -0.2989 | Yes |
| 36 | CELSR2 |  |  | 3378 | -0.475 | -0.2805 | Yes |
| 37 | SLC26A2 |  |  | 3382 | -0.476 | -0.2593 | Yes |
| 38 | OLFM1 |  |  | 3398 | -0.480 | -0.2409 | Yes |
| 39 | TFF3 |  |  | 3413 | -0.483 | -0.2222 | Yes |
| 40 | SLC29A1 |  |  | 3473 | -0.499 | -0.2143 | Yes |
| 41 | HOMER2 |  |  | 3531 | -0.521 | -0.2049 | Yes |
| 42 | CKB |  |  | 3572 | -0.536 | -0.1904 | Yes |
| 43 | FOS |  |  | 3621 | -0.561 | -0.1768 | Yes |
| 44 | CD9 |  |  | 3634 | -0.567 | -0.1537 | Yes |
| 45 | TPBG |  |  | 3638 | -0.570 | -0.1281 | Yes |
| 46 | PRSS23 |  |  | 3658 | -0.584 | -0.1059 | Yes |
| 47 | TOP2A |  |  | 3671 | -0.594 | -0.0815 | Yes |
| 48 | CA12 |  |  | 3694 | -0.611 | -0.0590 | Yes |
| 49 | NBL1 |  |  | 3743 | -0.664 | -0.0406 | Yes |
| 50 | ASCL1 |  |  | 3852 | -0.843 | -0.0295 | Yes |
| 51 | CXCL14 |  |  | 3892 | -1.060 | 0.0095 | Yes |
Table: GSEA details [plain text format]

  

Fig 2: HALLMARK\_ESTROGEN\_RESPONSE\_LATE: Random ES distribution      
 Gene set null distribution of ES for **HALLMARK\_ESTROGEN\_RESPONSE\_LATE**
